# Lung Cancer Screening Based on Type-different Sensor Arrays

**DOI:** 10.1038/s41598-017-02154-9

**Published:** 2017-05-16

**Authors:** Wang Li, Hongying Liu, Dandan Xie, Zichun He, Xititan Pi

**Affiliations:** 1 Key Laboratory of Biorheology Science and Technology, Ministry of Education, College of Bioengineering, Chongqing University, Chongqing, P.R. China; 2 Artificial Intelligence of Key Laboratory of Sichuan Province, Sichuan University of Science & Engineering, Zigong, Sichuan Province P.R. China; 3 Chongqing Engineering Research Center of Medical Electronics, Chongqing, P.R. China; 4 Chongqing Red Cross Hospital (People’s Hospital of Jiangbei District), Chongqing, P.R. China; 5 Key Laboratories for National Defense Science and Technology of Innovative Micro-Nano Devices and System Technology, Chongqing University, Chongqing, P.R. China

## Abstract

In recent years, electronic nose (e-nose) systems have become a focus method for diagnosing pulmonary diseases such as lung cancer. However, principles and patterns of sensor responses in traditional e-nose systems are relatively homogeneous. Less study has been focused on type-different sensor arrays. In this paper, we designed a miniature e-nose system using 14 gas sensors of four types and its subsequent analysis of 52 breath samples. To investigate the performance of this system in identifying and distinguishing lung cancer from other respiratory diseases and healthy controls, five feature extraction algorithms and two classifiers were adopted. Lastly, the influence of type-different sensors on the identification ability of e-nose systems was analyzed. Results indicate that when using the LDA fuzzy *5*-NN classification method, the sensitivity, specificity and accuracy of discriminating lung cancer patients from healthy controls with e-nose systems are 91.58%, 91.72% and 91.59%, respectively. Our findings also suggest that type-different sensors could significantly increase the diagnostic accuracy of e-nose systems. These results showed e-nose system proposed in this study was potentially practicable in lung cancer screening with a favorable performance. In addition, it is important for type-different sensors to be considered when developing e-nose systems.

## Introduction

In recent years, breath analysis has become a research focus in the field of respiratory disease diagnosis due to its noninvasiveness, convenience and real-time analysis^1^. The principal component in breath is water vapor, and the remaining parts include volatile organic compounds (VOCs) and nonvolatile matters dissolved in water or contained in exhaled aerosol particles^2^. To date, over 3000 different VOCs have been detected in human breath^3^, with some successfully used in the detection of diseases including lung cancer. VOCs in breath and their applications are shown in Table 1.

**Table 1:** VOCs in human breath.

Sample	Potential application	References
Carbon monoxide	Marker of neonatal jaundice	4
Hydrogen and methane	Gastrointestinal diagnoses	5, 6
Nitric oxide	Monitoring asthma therapy and COPD	7, 8
Ethanol	Potential indicator of nonalcoholic steatohepatitis, drunk driving test (law enforcement)	1, 9
Pentane	Marker of acute asthma, **lung cancer**, Rheumatoid arthritis, Pneumonia, alcoholic hepatitis, etc.	10–13
Acetone	Monitoring pneumonia and diagnosing Ketosis, diabetes, **lung cancer**, etc.	14–18
Hydrogen sulfide	Periodontal disease	19
Decane, 4-methy-octane, undecane, aldehydes, benzene and its derivatives, 1-butanol	Markers of **Lung cancer**	20–23
Methyl-mercaptan	Markers of Hepatic coma	24
Naphthalene, 1-methyl- and cyclohexane, 1,4-dimethyl-	Markers of pulmonary tuberculosis	25
Isoprene	Markers of advanced fibrosis in chronic liver disease and cholesterologenesis	26, 27
Carbonyl sulfide	Biomarkers of human liver disease and lung transplant recipients with acute rejection	28, 29
Carbon disulfide, pentane	Potential Markers of schizophrenia	30
Ammonia	Diagnosing chronic kidney disease, renal failure, hepatic encephalopathy, etc.	31–33

At present, a Tedlar^®^ bag is often used in sampling VOCs in breath^34^. Collected VOCs could be tested by multiple spectrometric techniques such as gas chromatography, ion transfer reaction, ion flow tube, ion mobility and so on^35, 36^. These analytical techniques are sensitive and accurate, but they also have many restrictions, such as high cost, requiring professional operation and requiring pre-concentration of the breath^37^. In recent years, inexpensive and portable e-nose systems have been proposed and designed for respiratory disease detection, the measurement reproducibility of the e-nose system was also validated to be acceptable^38^. The common methodology and application of e-nose systems were described in detail elsewhere^39, 40^. For lung cancer detection, Mazzone *et al*. reported an e-nose based on colorimetric sensor arrays could show a good performance^41, 42^; Haick’s team from the Israel Institute of Technology developed an e-nose system with gold nanoparticle sensors for the detection of lung cancers as well. Simulations revealed the accuracy for lung cancer detection to be over 86%, and subsequent experiments proved that this e-nose system could identify many types of cancers^20, 43^; Blatt *et al*. used metal oxide semiconductor sensor arrays for lung cancer diagnosis with accuracy, sensitivity and specificity all of over 90%^44^. However, these electronic noses are often based on sensor arrays with similar response principles. These sensors are very similar in terms of sensitivity and response patterns. Less research has been performed on type-different sensors for the diagnosis of lung cancers. Different feature extractions and classifiers could significantly affect recognition effects of sensor arrays, but little has been reported in this area.

In the current study, we used 14 gas sensors and 2 temperature/humidity sensors to develop a small-sized e-nose system. Samples from 52 volunteers were tested using the system. A software based on C# was also programmed to control detection process and generate the “breath pattern”. Five algorithms for extracting features from the “breath pattern” were compared. Finally, a 10 fold cross validation method was used to investigate the classification performance of the e-nose system with two classifiers. Lastly, whether type-different sensor arrays could improve the recognition ability of e-nose systems was also studied.

## Materials and Methods

### Selection of type-different sensors

According to the major components and their concentration ranges in human breath especially from lung cancer patients (Table 1), we selected 14 gas sensors which could be classified into 4 types: metal oxide semiconductor (MOS), hot wire gas, catalytic combustion gas, and electrochemical gas sensors.

None of the four classes of sensors are gas specific sensors; they are all cross response sensors, i.e. different gas sensors responding differently to the same gas mixture and the responses of the same sensor to different gas mixtures are also different. According to this response pattern, sensor arrays could form characteristic “breath fingerprints” by exhalation components, and could diagnose diseases through pattern recognition of a “breath fingerprint”. All sensors used in this study are commercial sensors, considering they are robust and stable. These sensors could be easily obtained to carry out repetitive tests. Details of gas sensors used in this study are listed in Table 2.

**Table 2:** Sensors used in this study.

No.	Model	Type	Range (ppm)	Detectable gases	Manufacturer
1	TGS2620	Metal oxide semiconductor	50–5000	Ethanol, hydrogen, butane, etc.	FIGARO
2	TGS2602	Metal oxide semiconductor	1–30	Toluene, hydrogen sulfide, ethanol, etc.	FIGARO
3	TGS2600	Metal oxide semiconductor	1–30	Hydrogen, ethanol, butane, etc.	FIGARO
4	TGS826	Metal oxide semiconductor	30–300	Ethanol, ammonia, hydrogen, etc.	FIGARO
5	TGS822	Metal oxide semiconductor	50–5000	Acetone, ethanol, benzene, etc.	FIGARO
6	TGS2444	Metal oxide semiconductor	10–300	Ammonia, hydrogen sulfide, ethanol, etc.	FIGARO
7	TGS8669	Metal oxide semiconductor	1–500	Acetone, benzene, toluene, etc.	FIGARO
8	WSP2110	Metal oxide semiconductor	1–50	Benzene, toluene, ethanol, etc.	Winsen
9	NAP-55A	catalytic combustion type gas sensor	500–5000	Combustible gases	NEMOTO
10	MR516	Hot-wire Gas Sensor	0–500	Formaldehyde and other VOCs	Winsen
11	ME3-C7H8	Electrochemical gas sensor	0–500	Toluene, xylene, Hydrogen sulfide, etc.	Winsen
12	ME4-C6H6	Electrochemical gas sensor	0–100	Benzene, xylene, toluene, etc.	Winsen
13	ME4-H2S	Electrochemical gas sensor	0–100	Hydrogen sulfide, hydrogen phosphide, formaldehyde, etc.	Winsen
14	CO-B4	Electrochemical gas sensor	0–50	Carbon monoxide	Alphasense

### E-nose system design

**Figure 1:**
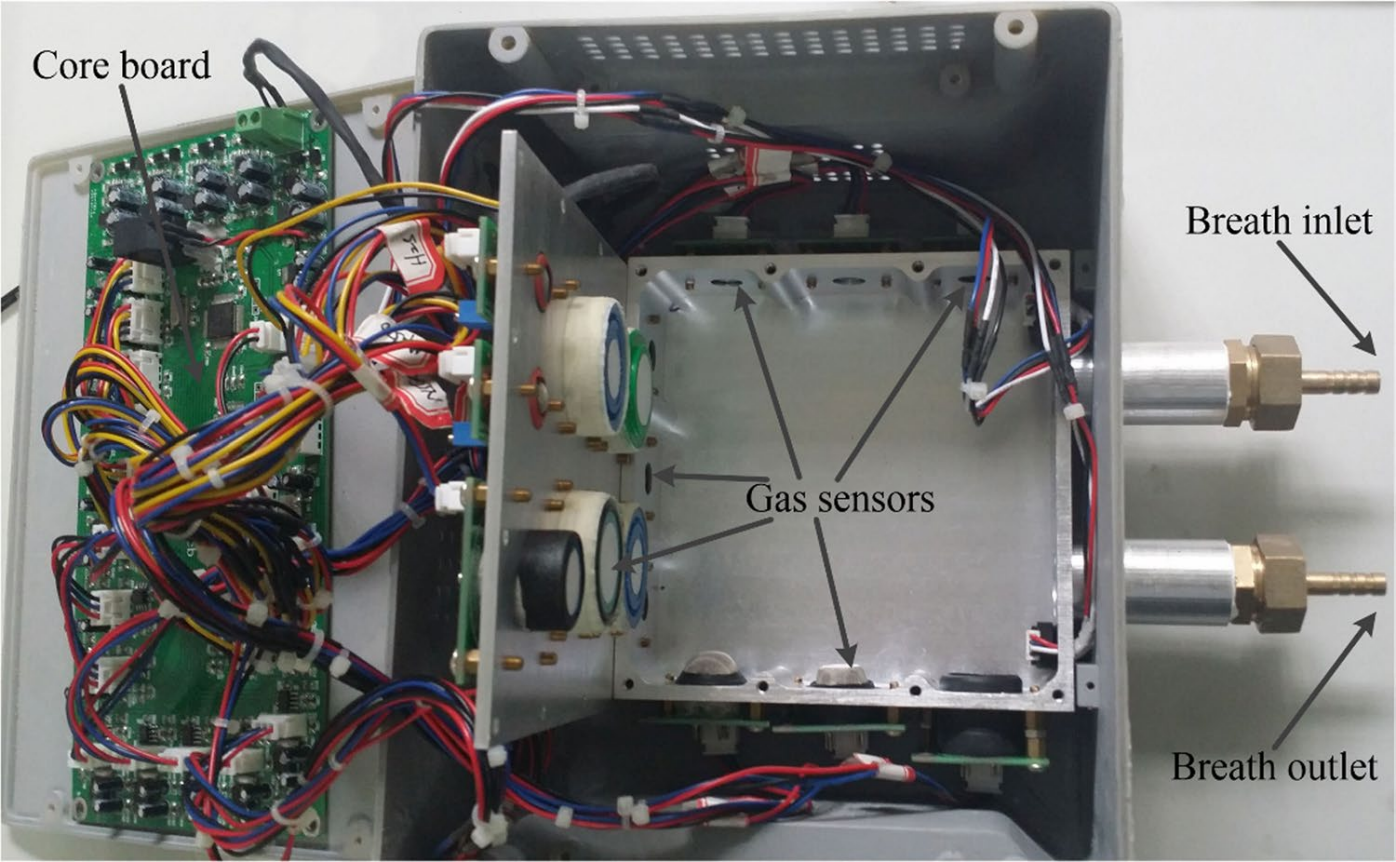
The photo of the designed E-nose system.

We designed the miniature e-nose system using the above mentioned sensors. The system’s gas reaction chamber is a rectangular enclosure made from aluminum alloy, with a volume of about 220 ml. All sensor probes are embedded into the gas reaction chamber. Sampling frequency of the whole system was designed to be 10 Hz. When the gas to be tested passes through the gas chamber, the sensor arrays will respond and form voltage (or current) signals. After initial processing (such as filtering, amplification, etc.), signals are sent to the main control chip (STM32F10) for analog digital conversion and temperature/humidity compensation calculations. Finally the signals are sent to the computer through a universal serial bus (USB) for display and storage. The compensation method of temperature/humidity involves taking the means of the two groups of temperature/humidity sensors (Model:HTG3515CH, Humirel Inc., France) placed at the air inlet and air outlet for linear compensation. The electronic nose system designed for this study is shown in Fig. 1.

In addition, in the current study, we programmed upper computer software based on C#. This software controlled detection modalities of the electronic nose system, as well as the display, storage of test data, and sample information management. The database used by this software was mySQL (Oracle^®^, CA, USA).

**Figure 2:**
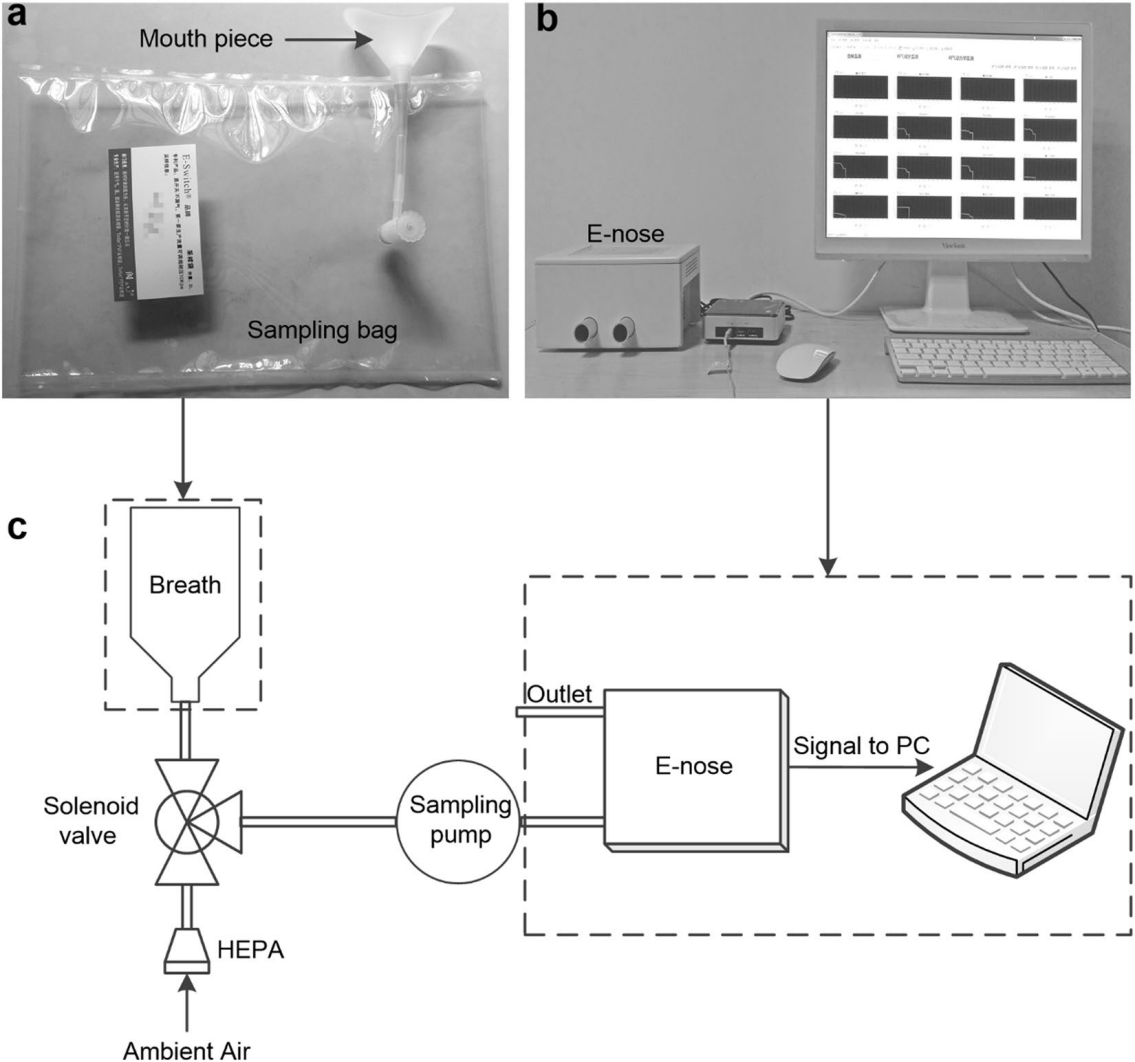
Overview of the breath sampling and analysis system. (**a**) Tedlar^®^ bag for breath sampling. (**b**) Photograph of the E-nose system and software interface. (**c**) Block diagram of the system.

Lastly, all breath samples in this study were collected by the 2 L Tedlar^®^ bag (E-switch^®^, China), and pumped into the designed e-nose system by a gas sampling pump. The detection platform is shown in Fig. 2.

### Source of test samples

Breath samples in this study included 24 cases from lung cancer patients, 5 cases from patients with other respiratory diseases (4 with COPD and 1 with Silicosis), 10 cases from healthy smokers and 13 healthy non-smokers. Among them, lung cancer patients were from the in-patient department of respiration at the Second Affiliated Hospital of Chongqing Medical University; patients with other respiratory diseases were from People’s Hospital of Jiangbei District and the Second Affiliated Hospital of Chongqing Medical University; healthy control volunteers (smokers and non-smokers) were recruited from Chongqing University. All volunteers singed informed consent after a detailed introduction of the purpose and plan of this study. Protocols including any relevant details of this study were carried out in accordance with the relevant guidelines and approved by Medical Ethics Committee of Chongqing University. Details of all volunteers for this study are listed in Table S1.

### Test process

All volunteers involved in this study fasted overnight prior to sampling. Meanwhile, smokers were asked to stop smoking two hours prior to sampling. All Tedlar^®^ bags were washed with nitrogen three times prior to sampling. The process for collection VOCs in breath is as follows:

1. All volunteers rested for 3–5 minutes and rinsed their mouths with clear water 3–5 times.
2. All volunteers put on nose clips and breathed in deeply with their mouths, exhaling into sampling bags via a disposable mouth piece until the bags were filled.
3. Sampling bags were then tightened and marked.

All samplings experiments were carried out in a well-ventilated room to avoid interference of other scents. All breath samples were tested within 8 hours after sampling; otherwise samples would be re-collected.

The breath detection process is as follows:

1. Opening the gas sampling pump to pump ambient air into the gas chamber at a rate of 6 L/min. Sensor response gradually stabilized near baseline, which normally took 30–80 s. This process can be termed a “preparation phase”.
2. Breath samples were collected and pumped into the gas chamber, and the sensors started to respond. This process took 10 s and can be termed the “ventilation phase”.
3. Disconnecting the gas sampling pump resulted in sensor responses gradually stabilizing. This process took 50 s and can be called the “response phase”.
4. Opening the gas sampling pump to inlet ambient air. Sensor response gradually returned to baseline. This process took 30 s and can be called “deflation phase”.

The effective detection period of each sample in this study includes a “ventilation phase”, a “response phase” and a “deflation phase”, altogether totaling 90 s. Each stage of the detection process was precisely controlled by a timing relay and electromagnetic valve. A high efficiency air filter was used when ambient air was pumped into the chamber during the “preparation phase” to dispose of solid debris that might exist in the air, avoiding sensor damage and ensuring baseline stability. All sample detections were carried out at the same place with good indoor ventilation.

### Data analysis

For successful identification of disease with an e-nose system, raw data should be analyzed and processed appropriately. All data was processed using Matlab. The data analysis procedure is as follows:

#### Data pre-processing

All raw data measured by the electronic nose system should be pre-processed before analysis, which includes baseline processing and standardization.

Baseline processing was performed for drift compensation and contrast enhancement^45^. Assuming a total of N_S_ samples were detected in this study, each sample contained N_D_ sensor response data; the length of each sensor detection data was N_T_; and the length of the stabilized data at the “preparation phase” was N_B_ (N_B_ ≤ N_T_); then the response (after baseline processing) to sample S (S = 1, 2, 3, …, N_S_) from sensor D (D = 1, 2, 3, …, N_D_) at time T (T = 1, 2, 3, …, N_T_) would be:

1 $${R}_{(S,D,T)}^{B}={R}_{(S,D,T)}-\frac{1}{{N}_{B}}\sum _{t=1}^{{N}_{B}}{R}_{(S,D,t)}$$

In which, R_(S, D, T)_ and R_(S, D, t)_ are the actual responses to sample S from sensor D at time T and time t (t = 1, 2, 3, …, N_B_).

Data standardization is necessary in order to compensate for the numerical range and/or unit differences of the results measured by sensors in the gas chamber^43^. A standard deviation method was adopted in this study for data standardization, i.e. the average response of each sensor was 0 and the standard deviation was 1 after standardization.

#### Feature extraction

In this study, the sampling frequency of electronic nose system was set at 10 Hz. The data dimension obtained from each sensor in one sampling cycle was 10 × 90 = 900. For each sample, there were actually 16 sensors for detection. Therefore, the feature vector dimension consisting of sensor responses obtained from each sample was 900 × 16 = 14400, far greater than the total number of samples included in this study. There was a great amount of redundancy in these dimension data. Redundant data were not only non-conducive to the internal parameters of the calculation classification method, but also would lead to over-fitting^46^. Therefore, it is necessary to reduce the dimension of the obtained characteristic matrix and extract main features. In the current study, we searched for optimal dimension reduction mapping from five different dimension reduction methods, including principal component analysis (PCA), linear discriminant analysis (LDA), Laplacian Eigenmap (LE), local linear embedding (LLE), and t-Stochastic Neighbor Embedding (tSNE).

When applying the features for classification after PCA dimension reduction, it was necessary to determine the number of principal components-*k*. The method used in this study was: the eigenvalues of the covariance matrix of the original data set were arrayed from large to small as: λ_1_, λ_2_, …, λ_n_, with the total variance percentage ***τ*** of the first *k* components occupied being:

2 $$\tau =\frac{{\sum }_{j=1}^{k}{\lambda }_{j}}{{\sum }_{j=1}^{n}{\lambda }_{j}}$$

We set ***τ*** = 99% in this study to calculate the parameter *k*.

#### Classifier selection

In this study, we initially selected 2 classifiers, *fuzzy k-NN* and support vector machine (*SVM*). Optimal values of near neighbor *k* and proportionality coefficient *m* in *Fuzzy k*-NN were determined using the 10-fold cross validation method. The kernel function used in SVM was a radial basis function. Penalty factor C and kernel function parameter σ were determined via grid search method^45^.

### Error estimation

When classifying the breath samples, 5 samples from other respiratory disease were excluded due to small sample size. Meanwhile, healthy smokers and healthy non-smokers were classified as the healthy group. The remaining 47 samples could be classified into the following two groups: lung cancer negative group and lung cancer positive group. The confusion matrix formed by real results and predicted results is shown as Table 3:

**Table 3:** Confusion matrix obtained from classifier.

		Predicted results	
		Positive	Negative
Real results	Positive	TP	FN
	Negative	FP	TN

Then the accuracy rate (Acr), sensitivity (Tpr) and specificity (Tnr) are:

3 $$Acr=\frac{TP+TN}{TP+FP+TN+FN}$$

4 $$Tpr=\frac{TP}{TP+FN}$$

5 $$Tnr=\frac{TN}{FP+TN}$$

### Data Availability

All data generated or analyzed during this study are included in this published article (and its Supplementary Information files).

## Results and Discussion

### Response curves of sensor arrays

**Figure 3:**
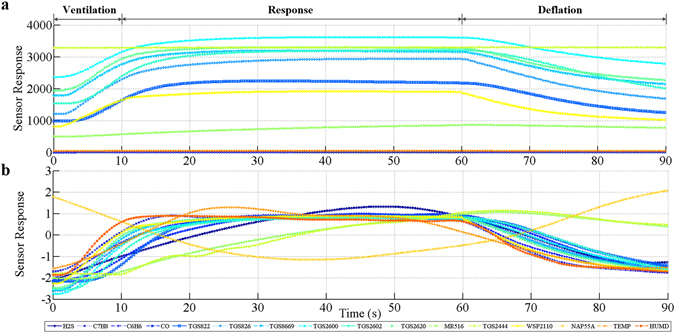
Typical response curves of the sensor arrays. (**a**) Response curves of the sensor arrays before preprocess. (**b**) Response curves of the sensor arrays after preprocess.

The typical response curve of the electronic nose system to the breath sample is shown in Fig. 3. It can be seen that the curve contains the three phases of one detection period, i.e.: ventilation phase, response phase and deflation phase. The response curves of most sensors were similar within the detection period. However, the curves of some sensors appeared to be quite different, such as TGS2444, MR516 and NAP55A. These pattern differences made the “breath fingerprint” more apparent.

### Comparison of feature extraction effects

**Figure 4:**
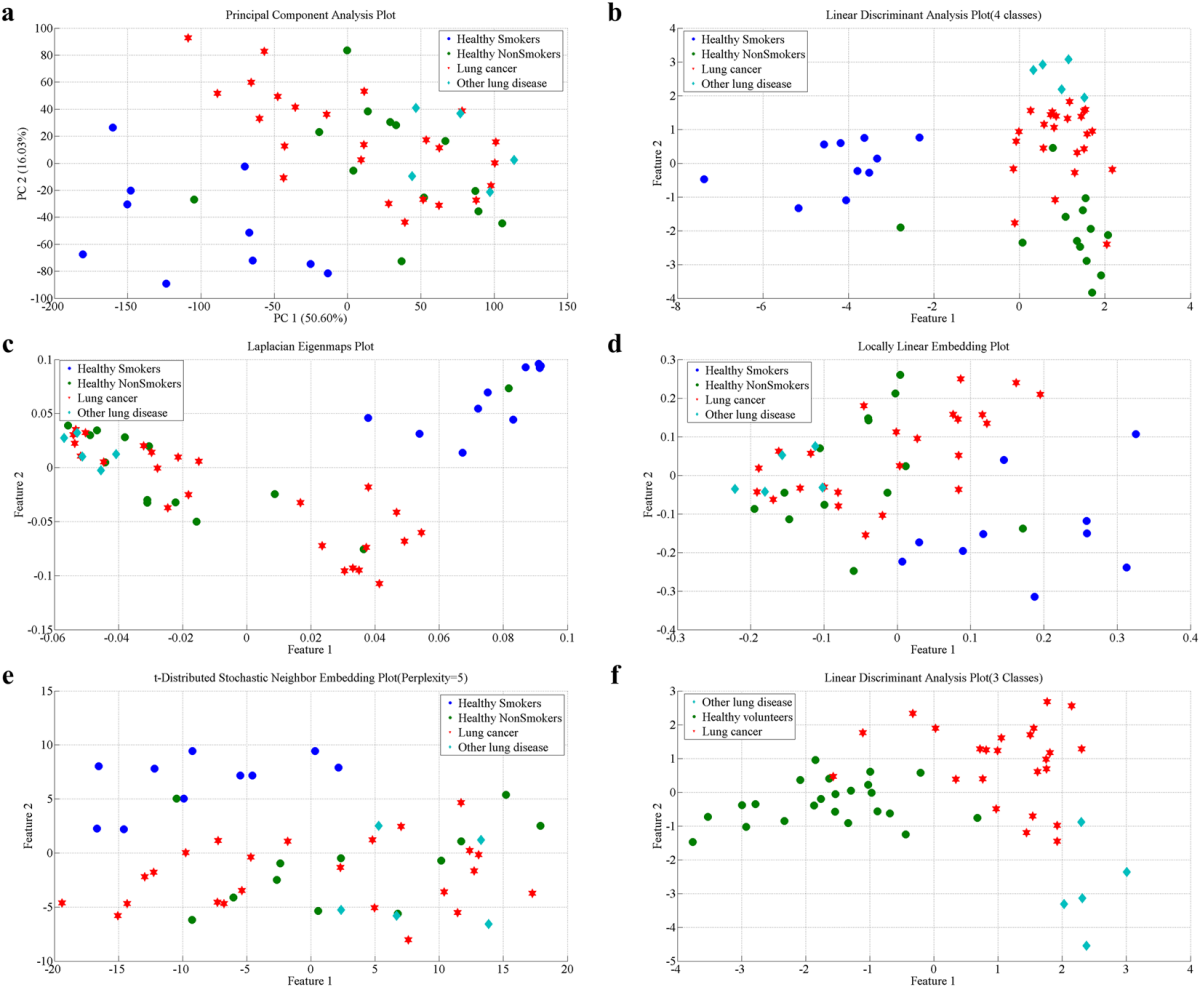
Mapping results of 5 algorithms (2D). (**a**) Represents the 2D mapping plot of PCA, (**b**) is the 2D mapping result of LDA with 4 classes of labels, **c** is the 2D mapping plot of LE, (**d**) is the 2D mapping plot of LLE, (**e**) is the 2D mapping result of tSNE, and (**f**) is the 2D mapping results of LDA with 3 classes of labels. Green dots represent samples from healthy non-smokers, blue dots represent samples from healthy smokers, red hexagrams represent samples from lung cancer patients, and light blue diamonds represent other disease samples.

When performing feature extraction, some methods involved parameter selection, such as the near neighbor number *k* in LE algorithm and the Perplexity parameter in the tSNE algorithm. We compared the LE mapping results of *k* = 3, 5, 7, 9, 11, finding the best one to be when *k* = 7. Similarly, after comparison, the Perplexity parameter in tSNE was set as 5. Besides, LDA is a supervised dimension reduction algorithm, such that the mapping results of different sample labels were different. In this study, we examined dimension reduction results via LDA for 4 types of sample labeling (lung cancer group, other respiratory disease group, healthy smoking group and healthy non-smoking group) and 3 types of labeling (healthy smokers and healthy non-smokers were classified together as a healthy group). The 2D mapping results of different feature extraction methods are as follows:

It can be seen in Fig. 4 (a–e) that different feature extraction methods may result in different classifications. However, we could also conclude from the 5 dimension reduction mapping results that:

First, in the above 5 kinds of feature extraction methods, LDA had the best classification performance for 4 types of sample labels in this study. This was indicated by the great distances between the 4 groups and the aggregation of samples in the same group as shown in Fig. 4b. In addition, there was little overlap between different groups. The next best classification results were from LE and PCA.

Second, no matter which dimension reduction algorithm was applied, healthy smokers and lung cancer patients could all be well classified, i.e. the blue dots and red hexagrams are mutually far away and have nearly no overlap in Fig. 4a–e. This also indicates that although many lung cancer patients in this study were also smokers or former smokers, their exhaled breath were significantly different from that of healthy smokers. This result was practically significant. Indeed, the smokers are a high risk population for lung cancer. However, the e-nose system used in this study could optimally distinguish lung cancer patients from healthy smokers in this high risk population. This also suggests that the simple screening of lung cancer patients in a high risk population is practically feasible.

Third, if samples were classified by the 3 classes of labels (healthy smokers and healthy non-smokers were classified as the healthy group), then we should only consider the dots (blue and green) as one group. This way, it can be seen that there is some overlap between the healthy group and lung cancer patients in the 2D mapping results of dimension reduction by PCA, LE, tSNE and LLE. However, Fig. 4f indicates that LDA still had a good classification performance for the 3 classes of sample labels.

Figure 4a–e reveals that no matter which dimension reduction method was adopted, the e-nose system developed in this study was able to distinguish healthy smokers from healthy non-smokers, as shown in Fig. 4; blue and green dots are far away from each other with only slight overlap. This result suggests that the breath components of smokers and non-smokers might be quite different, consistent with the conclusions of related studies^47^.

Lastly, Fig. 4 also showed that samples from other respiratory diseases (light blue diamonds) were all distinguishable from samples of healthy smokers (Figure a-e), from samples of the healthy population (Fig. 4f), and from samples of lung cancer patients (Fig. 4f). However, due to the insufficient sample size of patients with other respiratory diseases, this conclusion should be further validated with more samples.

### Estimation error analysis

As mentioned before, to carry out this part of study, we excluded samples from patients with other respiratory disease, and classified the remaining 47 samples into 2 groups: a healthy group and a lung cancer group. Besides, according to the previous dimension reduction comparison, data for classification were from feature extraction results by LDA, LE and PCA. When using the mapping results by PCA, according to the methods introduced before, the principal component number was 21, and these components explained 99.08% of the variance.

**Figure 5:**
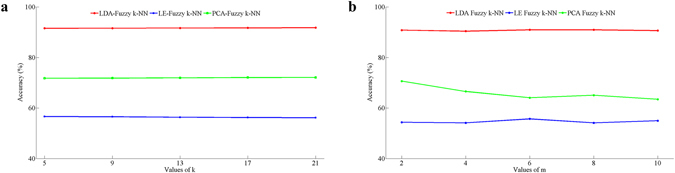
Optimization of *Fuzzy k-NN* algorithm parameters (*k, m*).

When using Fuzzy *k*-NN for classification, the near neighbor number *k* and the proportionality coefficient *m* should be optimized. A 10 fold cross validation method was used for optimization in this study; results are shown in Fig. 5. It can be seen in Fig. 5a that when *k* = 5, 9, 13, 17, 21, classification accuracy of *Fuzzy k-NN* algorithm remains unchanged. Therefore, k = 5 was utilized in this study. Figure 5b showed the effects of proportionality coefficient *m* on the classification accuracy of *Fuzzy 5-NN*. It can be seen that when *m* = 2, the classification accuracy of 3 classification method yielded relatively better results. Therefore, m was set as 2 in this study.

**Figure 6:**
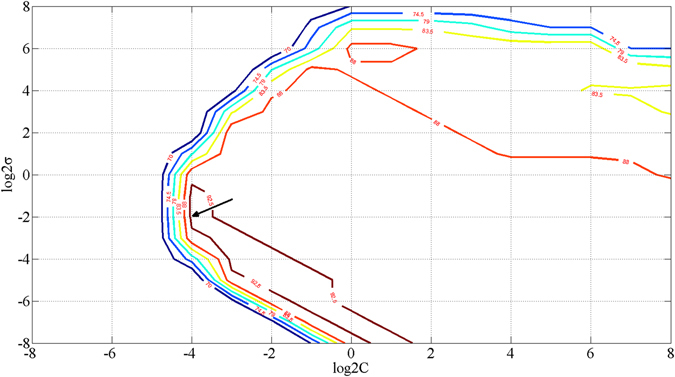
Contour map of PCA-SVM parameter (*C*, *σ*) optimizing. The arrow is pointing at the optimal parameter by cross validation of the grid optimization.

As described before, a grid search method was used to optimize the parameters (C, σ) of SVM^48^. Optimized results for PCA-SVM are shown in Fig. 6. When (C, σ) = (0.0625, 0.25), the classification accuracy of PCA-SVM is the best. Similarly, optimal (C, σ) of LDA-SVM and LE-SVM were also determined by this method.

After all parameters were optimized, 47 breath samples were classified using 2 classifiers based on the mapping results of 3 feature extraction methods mentioned above. Here, we also tested the classification performance by the 10-fold cross validation method. Each combined classification method was tested 50 times to obtain the confidence intervals of classification errors (shown in Table 4). It can be seen from the table that the best classification performance among the 6 classification methods were the combined methods based on LDA (LDA-Fuzzy *5*-NN and LDA-SVM). This was consistent with results observed in the dimension reduction mapping plots (Fig. 4). Among them, LDA *Fuzzy 5-*NN showed the best classification results, which produced greater than 90% of sensitivity, specificity and accuracy.

**Table 4:** Distinguishing results of lung cancer samples and healthy samples by different classification methods.

Classifier	Sensitivity[95% CI]	Specificity[95% CI]	Accuracy[95% CI]
LDA-Fuzzy *5*-NN	**91.58%** [90.01%, 93.15%]	**91.72%** [90.35%, 93.09%]	**91.59%** [90.56%, 92.63%]
LE-Fuzzy *5*-NN	57.22% [55.7%, 58.75%]	56.14% [53.82%, 58.46%]	56.63% [55.18%, 58.08%]
PCA-Fuzzy *5*-NN	86.25% [84.71%, 87.79%]	56.76% [55.11%, 58.42%]	71.81% [70.6%, 73.02%]
LDA-SVM	90.83% [88.99%, 92.68%]	84.20% [81.42%, 86.98%]	87.59% [86.2%, 88.97%]
LE-SVM	64.58% [61.82%, 67.35%]	55.07% [52.57%, 57.57%]	59.93% [58.35%, 61.51%]
PCA-SVM	57.64% [51.56%, 63.71%]	23.62% [20.39%, 26.86%]	40.99% [37.58%, 44.41%]

### Effects of type-different sensor array on lung cancer recognition

In order to study the effects of type-different sensor arrays of the e-nose on lung cancer recognition performance, we classified the 14 gas sensors into 2 groups: group K and group T (Table 5). Of them, group K included all 4 types of sensors, and group T included only MOS sensors. According to the response results (Fig. 1), the sensitivity, response patterns and principles of sensors in group K had a greater relative difference from each other than that of sensors in group T.

**Table 5:** Sensors grouping.

Group K		Group T	
Model	Type	Model	Type
ME3-C7H8	Electrochemical	TGS822	Metal oxide semiconductor
ME4-C6H6	Electrochemical	TGS826	Metal oxide semiconductor
CO-B4	Electrochemical	TGS8669	Metal oxide semiconductor
MR516	Hot wire	TGS2600	Metal oxide semiconductor
TGS2444	Metal oxide semiconductor	TGS2602	Metal oxide semiconductor
WSP2110	Metal oxide semiconductor	TGS2620	Metal oxide semiconductor
NAP-55A	Catalytic combustion	WSP2110	Metal oxide semiconductor

**Figure 7:**
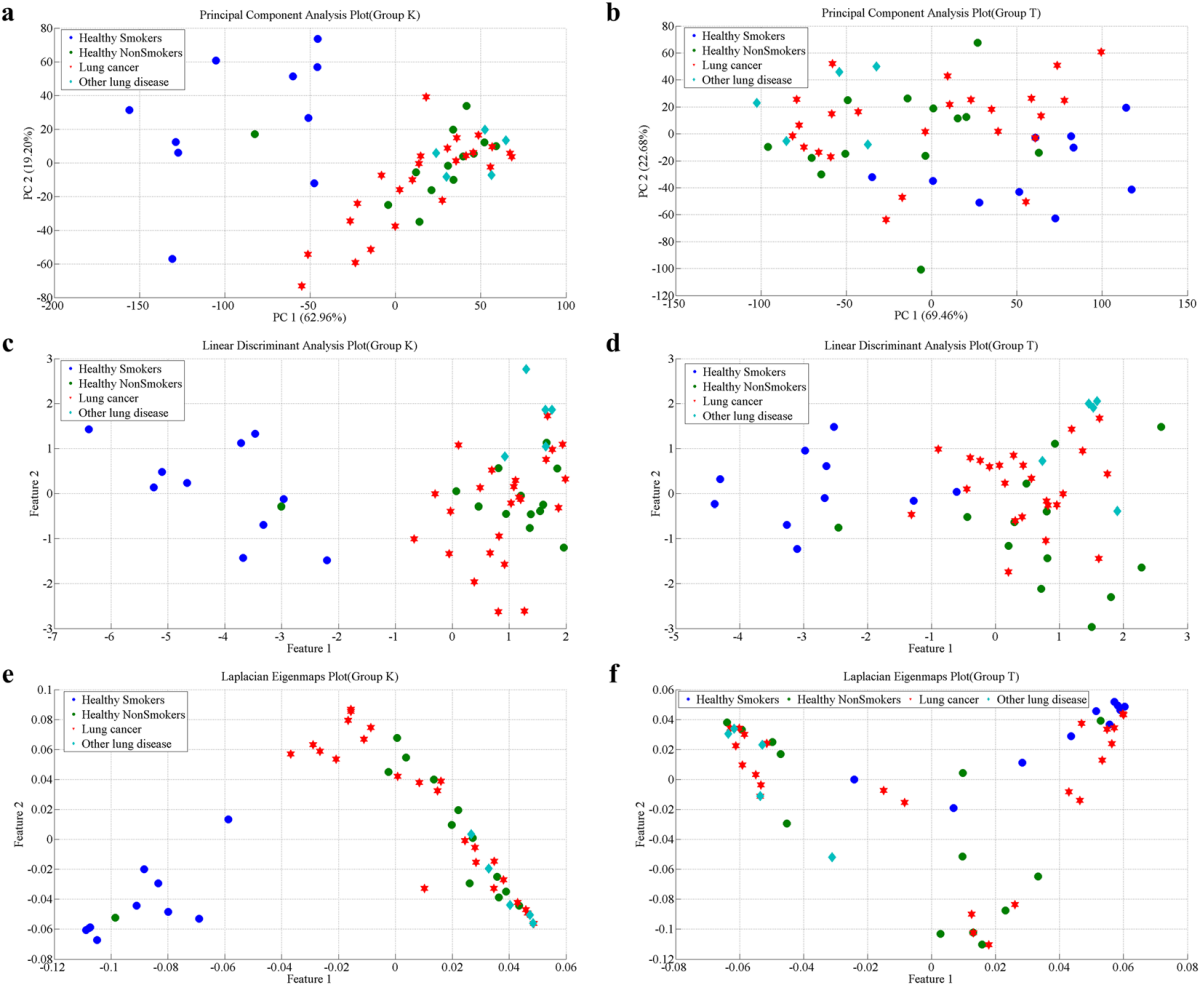
Mapping results of PCA, LDA and LE using data from sensor group T and group K. (**a**,**c**,**e)** are 2D mapping plots of PCA, LDA and LE based on data obtained by sensor group K; (**b**,**d**,**f**) are 2D mapping plots of PCA, LDA, and LE based on data obtained by sensors of group T.

We still used the 3 dimension reduction algorithms (LDA, PCA and LE) selected earlier to extract principal features of the data obtained by these 2 groups of sensors. For comparison, 52 samples were still classified into 4 groups (lung cancer group, healthy smoking group, healthy non-smoking group and other disease group). 2D plots of the mapping results are shown in Fig. 7. It can be seen that, no matter which group the data came from, the classification performance of all the 3 feature extraction methods decreased when comparing to the mapping results in Fig. 4. However, data from sensor group K gave better classification results (Fig. 7a,c,e) than that from sensor group T (Fig. 7b,d,f) when using the same feature extraction algorithm.

**Figure 8:**
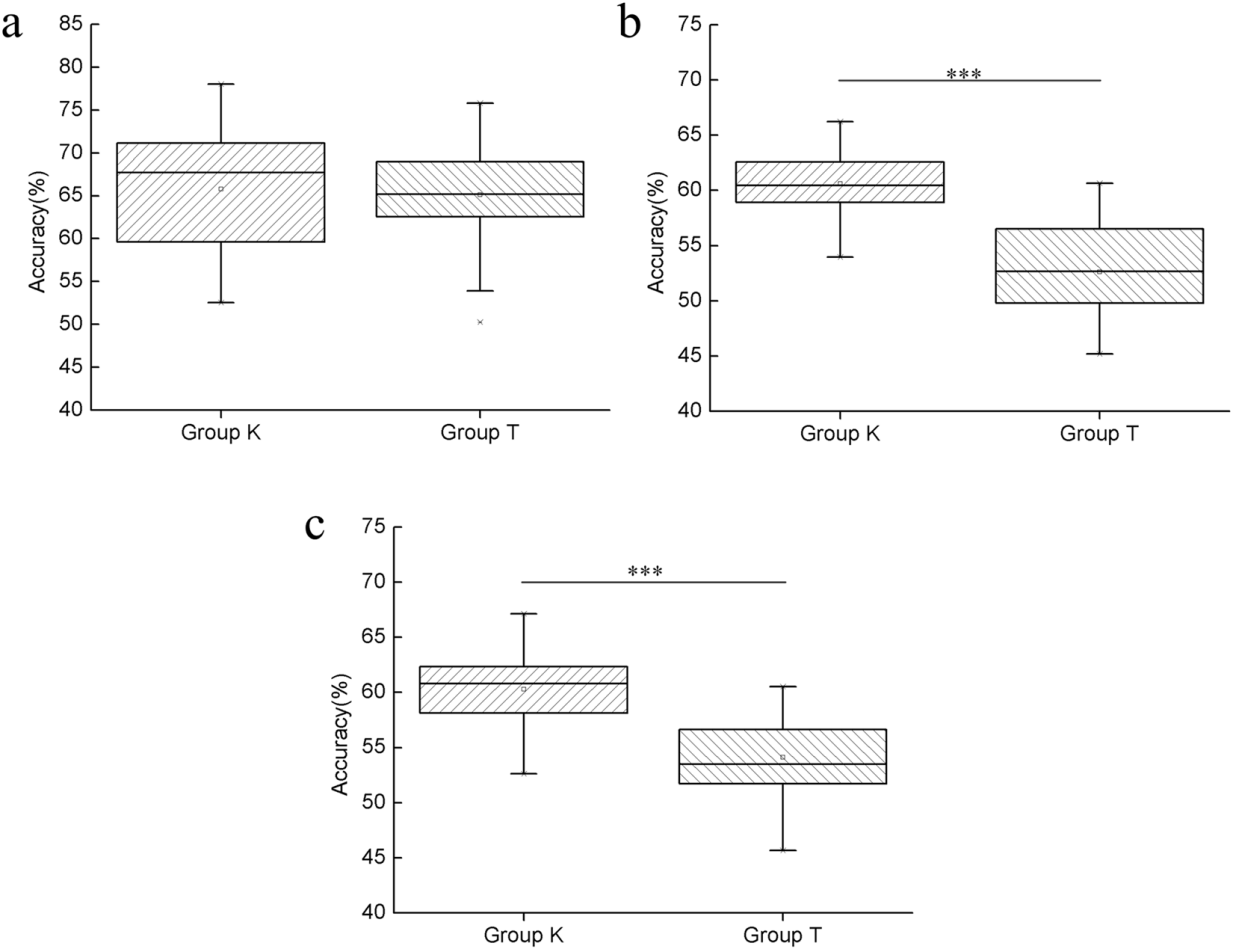
Accuracy of 3 classification methods based on the data from sensor group K and sensor group T. (**a**) Comparison of classification accuracy using LDA *5*-NN based on data from sensor groups K and T; (**b**) Comparison of classification accuracy using PCA *5*-NN based on data from sensor groups K and T; (**c**) Comparison of classification accuracy using LE *5*-NN based on data from sensor groups K and T. ***indicates significance: p < 0.01.

In order to further compare the recognition performance of sensor group K and sensor group T, we used the *Fuzzy k*-NN, which had better performance in the previous classification to classify breath samples based on mapping results depicted in Fig. 7. Similarly, for comparison, breath samples of other diseases were also excluded, and healthy smokers and non-smokers were classified as one group, i.e. 47 samples were divided into two groups: a healthy group and a lung cancer group. After parameter optimization, accuracy of 3 classification methods (LDA *5*-NN, PCA *5*-NN and LE *5*-NN) were obtained using 10-fold cross validation. We ran the validation 50 times to get 50 accurate readings for each classification method. Then, the recognition accuracies of sensor group K and sensor group T were analyzed using one way-ANOVA; results are shown in Fig. 8.

Similar conclusions could be drawn from results shown in Fig. 8 as in Fig. 7. Firstly, no matter which sensor group was used, the lung cancer recognition performance was not as good as the performance that all 14 gas sensors showed. Secondly, lung cancer detection accuracy was better when using data from sensor group K than that from sensor group T. When utilizing PCA *5*-NN (Fig. 8b) and LE 5-NN (Fig. 8c), the recognition accuracy of sensor group K was significantly greater than that of sensor group T (n = 50, p < 0.01). Although the classification accuracy of sensor groups K and T was not significantly different (n = 50, p = 0.21) when using LDA 5-NN, the average and highest recognition accuracy of sensor group K was greater than that of sensor group T after 50 times of cross validation. In summary, type-different sensors are notably helpful for improving the lung cancer recognition ability of the e-nose system designed in this study.

## Conclusion

The e-nose system developed using 4 types of commercial sensors in this study could identify relatively specific “breath fingerprints” based on human breath, which could be used to recognize volunteers in different diseased or healthy states. LDA proved to be among the best methods for “breath fingerprint” recognition in this study. When this e-nose system was used in the differentiation of lung cancer patients from healthy volunteers, the classification specificity, sensitivity and accuracy as determined by LDA fuzzy *5*-NN were all above 90%, indicating a comparable performance with traditional imaging modalities. In addition, difference in the type of sensor arrays are notably helpful for the improvement of the ability of the e-nose system to detect respiratory diseases.

In a word, the designed e-nose system based on optimized algorithms was low cost, noninvasive and was potential practicable in screening lung cancers from both healthy people and lung cancer high risk populations.

## Electronic supplementary material

Supporting Information
