# Supplementary material for: Lung Cancer Screening Based on Type-different Sensor Arrays
Source: Sci Rep. 2017 May 16;7:1969. doi: 10.1038/s41598-017-02154-9 (PMC5434050; doi:10.1038/s41598-017-02154-9)
Supplement: Supplementary file 1 — Supporting Information [file 41598_2017_2154_MOESM1_ESM.pdf]

Lung Cancer Screening Based on Type-different Sensor Arrays

Wang Li[1,2], Hongying Liu[1,3,*], Dandan Xie[1], Zichun He[4] and Xititan Pi[1,5,†]

[1]Key Laboratory of Biorheology Science and Technology, Ministry of Education, College of Bioengineering, Chongqing University, Chongqing, PR China

[2]Institute of Biomedical Engineering and Health Sciences, Changzhou University, Changzhou, Jiangsu Province, China

[3]Chongqing Engineering Research Center of Medical Electronics, Chongqing, PR China

[4]Chongqing Red Cross Hospital (People's Hospital of Jiangbei District), Chongqing, PR China

[5]Key Laboratories for National Defense Science and Technology of Innovative Micro-Nano Devices and System Technology, Chongqing University, Chongqing, PR China

*corresponding author. liuhongying@cqu.edu.cn

†corresponding author. pixitian@cqu.edu.cn

Table S-1 Details of volunteers

No.	Group	Chemical therapy	Age	Sex	Smoking amounts	Smoking history	Details of disease	Taking medication	Hereditary history	Pollutants exposure history
1	Lung cancer	NO	51	Male	1.5 packs/day	30	Squamous carcinoma	YES	NO	YES
2	Lung cancer	YES	62	Male	0.5 packs/day	40	Small cell lung cancer	YES	NO	NO
3	Lung cancer	YES	73	Male	EX-2-3 packs/day	10	Small cell lung cancer	YES	NO	NO
4	Lung cancer	YES	45	Male	NO	N/A	Squamous carcinoma	YES	NO	NO
5	Lung cancer	YES	66	Male	2 packs/day	50	Small cell lung cancer	YES	NO	YES
6	Lung cancer	YES	64	Male	0.5 packs/day	50	Adenomatous carcinoma	YES	NO	YES
7	Lung cancer	YES	70	Male	NO	N/A	Adenomatous carcinoma	YES	NO	YES
8	Lung cancer	YES	79	Female	NO	N/A	Adenomatous carcinoma	YES	NO	YES
9	Lung cancer	YES	63	Male	NO	N/A	Small cell lung cancer	YES	NO	YES
10	Lung cancer	YES	63	Male	1.5 packs/day	50	Small cell lung cancer	YES	NO	NO
11	Lung cancer	YES	59	Male	EX-1 packs/day	20	Adenomatous carcinoma	YES	NO	YES
12	Lung cancer	YES	58	Male	1.5 packs/day	40	Small cell lung cancer	YES	NO	YES
13	Lung cancer	NO	62	Male	1 packs/day	40	Squamous carcinoma	YES	NO	NO
14	Lung cancer	NO	79	Male	EX-1 packs/day	50	Adenomatous carcinoma	YES	NO	NO
15	Lung cancer	NO	64	Male	2 packs/day	40	Squamous carcinoma	YES	NO	YES
16	Lung cancer	NO	53	Male	0.5 packs/day	30	lung cancer	YES	NO	YES
17	Lung cancer	NO	53	Female	NO	N/A	Adenomatous carcinoma	YES	NO	NO
18	Lung cancer	YES	69	Male	EX-1 packs/day	40	Adenomatous carcinoma	YES	NO	NO

continued

No.	Group	Chemical therapy	Age	Sex	Smoking amounts	Smoking history	Details of disease	Taking medication	Hereditary history	Pollutants exposure history
19	Lung cancer	YES	70	Male	EX-2 packs/day	50	Large cell neuroendocrine lung cancer	YES	NO	NO
20	Lung cancer	YES	73	Male	1 packs/day	50	Adenomatous carcinoma	YES	NO	NO
21	Lung cancer	YES	69	Male	3 packs/day	40	Squamous carcinoma	YES	NO	NO
22	Lung cancer	NO	64	Male	1.5 packs/day	50	Squamous carcinoma	YES	NO	NO
23	Lung cancer	YES	66	Male	1packs/day	40	Small cell lung cancer	YES	NO	NO
24	Lung cancer	YES	51	Male	EX-1packs/day	20	Adenomatous carcinoma	YES	NO	NO
25	Other lung disease	NO	85	Male	EX-0.5packs/day	10	COPD[#]	YES	NO	NO
26	Other lung disease	NO	89	Male	EX-0.5packs/day	30	COPD	YES	NO	NO
27	Other lung disease	NO	80	Female	NO	N/A	COPD	YES	NO	YES
28	Other lung disease	NO	64	Male	2 packs/day	46	COPD	YES	NO	NO
29	Other lung disease	NO	67	Male	EX-2packs/day	30	Silicosis	YES	NO	YES
30	Healthy smokers	N/A[‡]	56	Male	1 packs/day	35	N/A	N/A	NO	YES
31	Healthy smokers	N/A	52	Male	1.5 packs/day	30	N/A	N/A	NO	NO
32	Healthy smokers	N/A	59	Male	1 packs/day	34	N/A	N/A	NO	YES
33	Healthy smokers	N/A	58	Male	1.5 packs/day	30	N/A	N/A	NO	NO
34	Healthy smokers	N/A	47	Male	1 packs/day	28	N/A	N/A	NO	NO
35	Healthy smokers	N/A	42	Male	1.5 packs/day	35	N/A	N/A	NO	NO
36	Healthy smokers	N/A	60	Male	1 packs/day	40	N/A	N/A	NO	NO
37	Healthy smokers	N/A	65	Male	1 packs/day	40	N/A	N/A	NO	NO
38	Healthy smokers	N/A	60	Male	2 packs/day	15	N/A	N/A	NO	NO
39	Healthy smokers	N/A	59	Male	1.5 packs/day	15	N/A	N/A	NO	NO
40	Healthy nonsmokers	N/A	49	Male	NO	N/A	N/A	N/A	NO	NO
41	Healthy nonsmokers	N/A	52	Male	NO	N/A	N/A	N/A	NO	NO
42	Healthy nonsmokers	N/A	53	Male	NO	N/A	N/A	N/A	NO	NO
43	Healthy nonsmokers	N/A	46	Male	NO	N/A	N/A	N/A	NO	NO
44	Healthy nonsmokers	N/A	26	Male	NO	N/A	N/A	N/A	NO	NO
45	Healthy nonsmokers	N/A	22	Male	NO	N/A	N/A	N/A	NO	NO
46	Healthy nonsmokers	N/A	23	Female	NO	N/A	N/A	N/A	NO	NO

continued

No.	Group	Chemical therapy	Age	Sex	Smoking amounts	Smoking history	Details of disease	Taking medication	Hereditary history	Pollutants exposure history
47	Healthy nonsmokers	N/A	25	Male	NO	N/A	N/A	N/A	NO	NO
48	Healthy nonsmokers	N/A	29	Male	NO	N/A	N/A	N/A	NO	NO
49	Healthy nonsmokers	N/A	23	Female	NO	N/A	N/A	N/A	NO	NO
50	Healthy nonsmokers	N/A	6	Male	NO	N/A	N/A	N/A	NO	NO
51	Healthy nonsmokers	N/A	34	Female	NO	N/A	N/A	N/A	NO	NO
52	Healthy nonsmokers	N/A	18	Male	NO	N/A	N/A	N/A	NO	NO

‡N/A:Not applicable

#COPD:Chronic Obstructive Pulmonary Disease
